# An Efficient 3D Cell Culture Method on Biomimetic Nanostructured Grids

**DOI:** 10.1371/journal.pone.0072936

**Published:** 2013-09-02

**Authors:** Maria Wolun-Cholewa, Krzysztof Langer, Krzysztof Szymanowski, Aleksandra Glodek, Anna Jankowska, Wojciech Warchol, Jerzy Langer

**Affiliations:** 1 Department of Cell Biology, Poznan University of Medical Science, Poznan, Poland; 2 Laboratory for Materials Physicochemistry and Nanotechnology, Adam Mickiewicz University, Srem, Poland; 3 Department of Mother's and Child's Health, Poznan University of Medical Science, Poznan, Poland; 4 Department of Biophysics, Poznan University of Medical Science, Poznan, Poland; Brandeis University, United States of America

## Abstract

Current techniques of *in vitro* cell cultures are able to mimic the *in vivo* environment only to a limited extent, as they enable cells to grow only in two dimensions. Therefore cell culture approaches should rely on scaffolds that provide support comparable to the extracellular matrix. Here we demonstrate the advantages of novel nanostructured three-dimensional grids fabricated using electro-spinning technique, as scaffolds for cultures of neoplastic cells. The results of the study show that the fibers allow for a dynamic growth of HeLa cells, which form multi-layer structures of symmetrical and spherical character. This indicates that the applied scaffolds are nontoxic and allow proper flow of oxygen, nutrients, and growth factors. In addition, grids have been proven to be useful in *in situ* examination of cells ultrastructure.

## Introduction

Cultures of human or animal cells in *in vitro* conditions are usually performed for their identification, proliferation or cell death assessment. Frequently, initial evaluations are carried out in two-dimensional (2D) cell culture systems. Despite their widespread use, observations made in 2D systems do not correspond to the results of *in vivo* studies [1]. Thus, currently, researchers are using three dimensional (3D) cultures that mimic the *in vivo* environment more accurately [2]. Cells grown in three-dimensional cultures are more valid targets for discovering and testing of new drugs for cancer treatment [3]. Contrary to 2D culture systems, 3D *in vitro* models have the potential to provide insights into cellular functions such as: differentiation, migration, and gene expression in a controlled and well-defined manner [4]–[6]. 3D cell culture main feature is the ability to mimic the extracellular matrix (ECM) conditions. Materials used for 3D cell culture systems production include both natural and synthetic biopolymers. Lately also biodegradable materials were introduced in scaffold construction, however it was proven that their stability in liquid environment is limited [5], [7]–[14].

One of the methods for fiber-based 3D scaffolds production is electro-spinning [9]. An advantage of those nanostructured grids, which differentiates them from other types of scaffolds, is the reduced diameter of pore size [15]–[16]. Moreover, electrospun scaffolds are built from very small fibers which create large surface areas [1], [9], [13], [15]. This allows for more accurate evaluation of cell proliferation [9], [11], [17].

In this study, electrospun nanostructured fibers in the form of spatial nanostructured 3D grids, fabricated from a polymer mixture, including polyaniline, were examined as a potential tool for 3D culture *in vitro*.

## Materials and Methods

### Synthesis of Polyaniline

6.9 g of aniline hydrochloride was dissolved in 300 ml of 1 M HCl. The solution of aniline hydrochloride was combined with a solution of 11.4 g ammonium persulphate dissolved in 200 ml of 1 M HCl. The mixture was left at room temperature for 12 hours with continuous mixing. Next the product was filtered, washed with water, methanol and chloroform. Polyaniline (PANI) was transferred into 200 ml of chloroform and dispersed to a suspension using ultrasound bath.

### Fabrication of Nanofibers using Electro-spinning

150 mg of polystyrene was dissolved in 2 ml of chloroform, gradually mixed with 50 mg of poly(ethylene oxide) – PEO with continuous mixing. Subsequently, the polystyrene and PEO were mixed with each other and supplemented with 8 ml of the previously obtained polyaniline – PANI suspension (protects against microbiological contamination), with mixing in order to obtain a uniform suspension of polyaniline in the solution of polymers. The process of electro-spinning was conducted in a non-uniform electric field under the voltage of 4.5 kV, inter-electrode distance of 15 cm and polymer solution outflow rate of 0.2 mL/min. Obtained nano- and microfibre random networks were transferred from the electrode into plastic carrier frames, forming final grids.

All reagents used for the synthesis of polyaniline and fabrication of nanofibers using electro-spinning were purchased from Sigma-Aldrich, St Louis, MO, USA.

### Cell Culture

Human HeLa cervical epithelial cells (ATCC CCL-2) were purchased from the American Type Cell Culture (ATCC, Manassas, MA). HeLa cells were cultured in standard conditions: RPMI medium (PAN Biotech-Gmbh, Aidenbach, Germany) supplemented with 10% fetal calf serum (FCS; Sigma, St Louis, MO, USA), 2 mmol/L L-glutamine (Cambrex, Charles City, IA, USA), 100 IU/mL penicillin and streptomycin solution (Sigma-Aldrich, St Louis, MO, USA) in a 5% CO2-humidified atmosphere at 37°C.

In order to examine the usefulness of nanostructural grids as a tool for 3D cultures, the cells were seeded on grids at a density of 10 000 cells. Subsequently, the grids with cells were placed into fresh culture medium and after 24, 48 or 72 hours cells morphology and their viability were examined. Every experiment was repeated ten times.

### Cell Viability Assessment

The viability of HeLa cells was analyzed using the XTT colorimetric test, based on the reduction of XTT compound (tetrazoline-2,3-bis(2-methoxy-4-nitro-5-sulphophenyl)-2H-5-carboxyanilide, Sigma-Aldrich, St Louis, MO, USA) by living cells. Optical density of colour product was measured at the wavelength of 450 nm.

The percentage of mitochondrial activity was calculated according to the following equation: (OD of nanostructured grid with cells−OD of medium alone)/(OD of nanostructured grid only−OD medium alone)×100; where OD is optical density [18]. Statistical analysis of the results was done with the Kruskal-Wallis test with Dunn’s post-test using STATISTICA ver.5 software (Statsoft, Krakow, Poland). *P* value less than 0.05 was considered statistically significant.

### Microscopy

#### Fluorescence microscopy

After 24, 48 or 72 hours of culture on the grids HeLa cells were stained with 0.1 µg/ml of Hoechst 33342 and 0.125 µg/ml of propidium iodide (Sigma-Aldrich, St Louis, MO, USA). The presence of intact, apoptotic and/or necrotic cells was evaluated using Nicon Diaphot Eclipse TE 200 fluorescence microscope, equipped with UV-2A and FITC/FLUO-3 filters [19].

#### Confocal microscopy

In order to visualize the 3D construct the cells were pre-fixed for 10 minutes in 4% paraformaldehyde (Sigma-Aldrich, St Louis, MO, USA) and stained with 0.125 µg/ml of propidium iodide (Sigma-Aldrich, St Louis, MO, USA). Signals were excited at 543 nm wavelength and fluorescence emission was selected with 560 nm bandpass filter. Images of cells were represented as orthogonal projections of 20–60 optical sections in 0.3 µm increments using Zeiss LSM 510 confocal microscope.

#### Scanning electron microscopy

After 48 hours of culture on the grids the cells were fixed in a standard way using 2.5% glutaraldehyde (at room temperature for 60 min and, then at 4°C for 24 hours). The grids with cells were washed with PBS for 30 min and the cells were gradually dehydrated in alcohols. The grids with cells were finally coated with palladium and visualized with a scanning electron microscope (Philips SEM 515).

#### Transmission electron microscopy

After 48 hours the nanostructured grids with the cells were put into a fixative solution containing 4% glutaraldehyde (Taab, Berkshire, UK), buffered with 0.1 M phosphate buffer, pH 7.3 (Merck). After overnight incubation the nanostructured grids were rinsed for 60 min in 0.1 M phosphate buffer, pH 7.3, and then postfixed in 2% OsO_4_ in 0.1 M phosphate buffer for 2 hours (Merck). The specimens were dehydrated in ethanol, block-stained with alcoholic uranyl acetate and embedded in Spurr’s medium (Merck). After contrasting, the ultrastructure of HeLa cells was examined using JEOL 100 Transmission Electron Microscope.

## Results

### Morphology of Polyaniline Fibers

**Figure 1:**
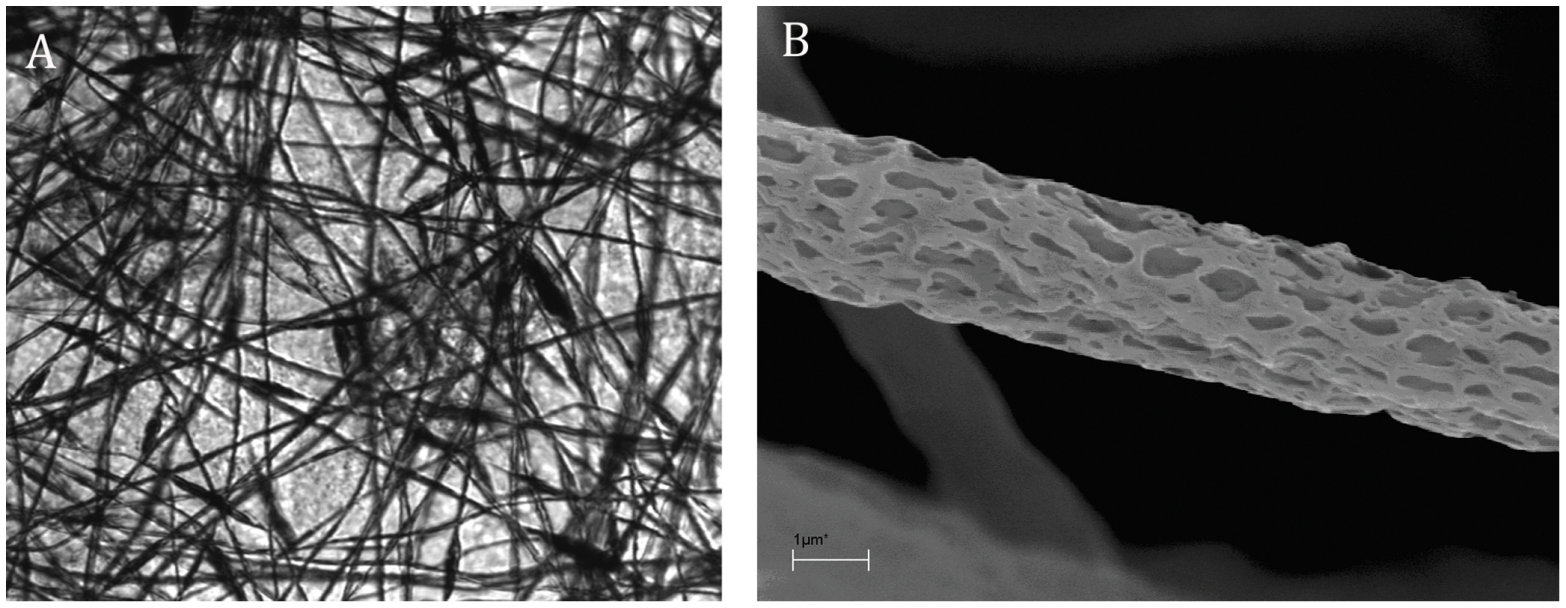
Light microscopy image of nanostructured grid. Original magnification x200 (A). Scanning electron microscopy image of nanostructured microfiber. Original magnification x500 (B).

Light microscopy confirmed that the analyzed grids are transparent (Fig. 1A). Moreover, scanning electron microscopy showed that the thickness of electrospun fibers was 2000–2500 nm, and the diameter of pores was 50–300 nm (shorter dimension). The average thickness of a grid is less than 0.5 mm (Fig. 1B).

### Cell Viability

To determine whether the tested electrospun scaffolds can influence the viability of uterine cervix carcinoma cells cultured in 3D, their viability was examined using the XTT assay. Due to XTT-reducing properties of the nanostructured grids alone, the viability of cells grown on the grids were related to the results obtained for measurements of the grids alone.

**Figure 2:**
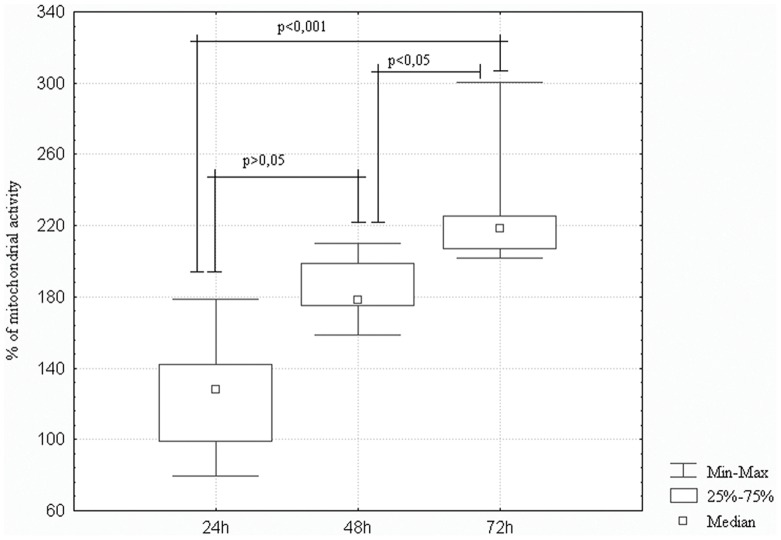
The viability of HeLa cells cultured on the nanostructured grid. HeLa cells were incubated for 24, 48 72 hours on the nanostructured grids and the percentage of mitochondrial activity was calculated according to the following formula: (OD of nanostructured grid with cells−OD of medium alone)/(OD of nanostructured grid only−OD medium alone)×100; where OD is optical density.

As shown in Fig. 2 the grids have no cytotoxic effect on HeLa cells. After 48 hours of culture on the nanostructured grids cell viability increased compared to the evaluation made after 24 hours. However, the change was insignificant (p>0.05). Subsequent incubation, up to 72 hours, resulted in a significant increase of cell viability, compared to the viability noted after either 24 hour (p<0.001) or 48 hour of culture (p<0.05) (Fig. 2).

### Cell Morphology

**Figure 3:**
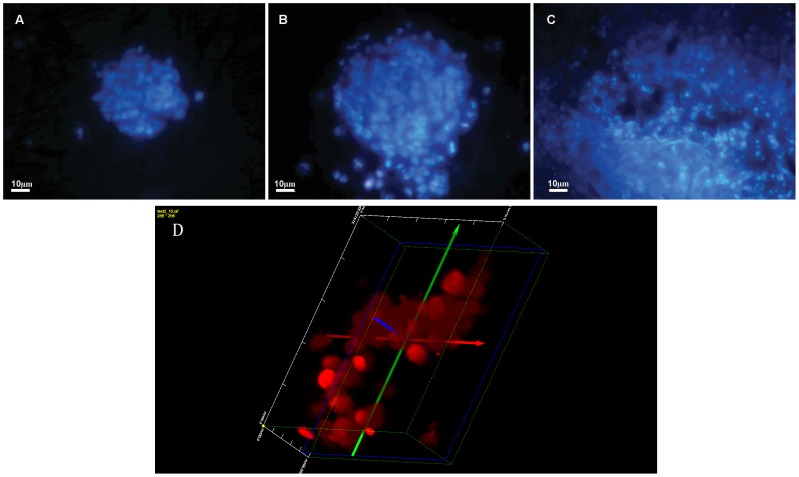
HeLa cells stained with Hoechst 33342 and propidium iodide after 24 (A), 48 (B) and 72 (C) hours of 3D culture on the nanostructure grids. Original magnification x200. A fragment of the spherical 3D structure of cell growing on nanostructured grids visualized after 48 hours. Original magnification x400 (D).

The double staining using Hoechst 33342 and propidium iodide allows distinguishing living and necrotic cells as propidium iodide penetrates only cells with a disrupted cell membrane, while Hoechst 33342 penetrates also intact cell membranes. As it was shown in Fig. 3 the nuclei of cells cultured on the nanostructured grids 24 (Fig. 3A), 48 (Fig. 3B) and 72 (Fig. 3C) hours emitted bright blue fluorescence. The staining proved that in the analyzed time intervals cells showed high viability with very low level of necrosis. Moreover, confocal microscopy confirmed that the cells cultured on the grids formed a homogenous 3D constructs (Fig. 3D).

**Figure 4:**
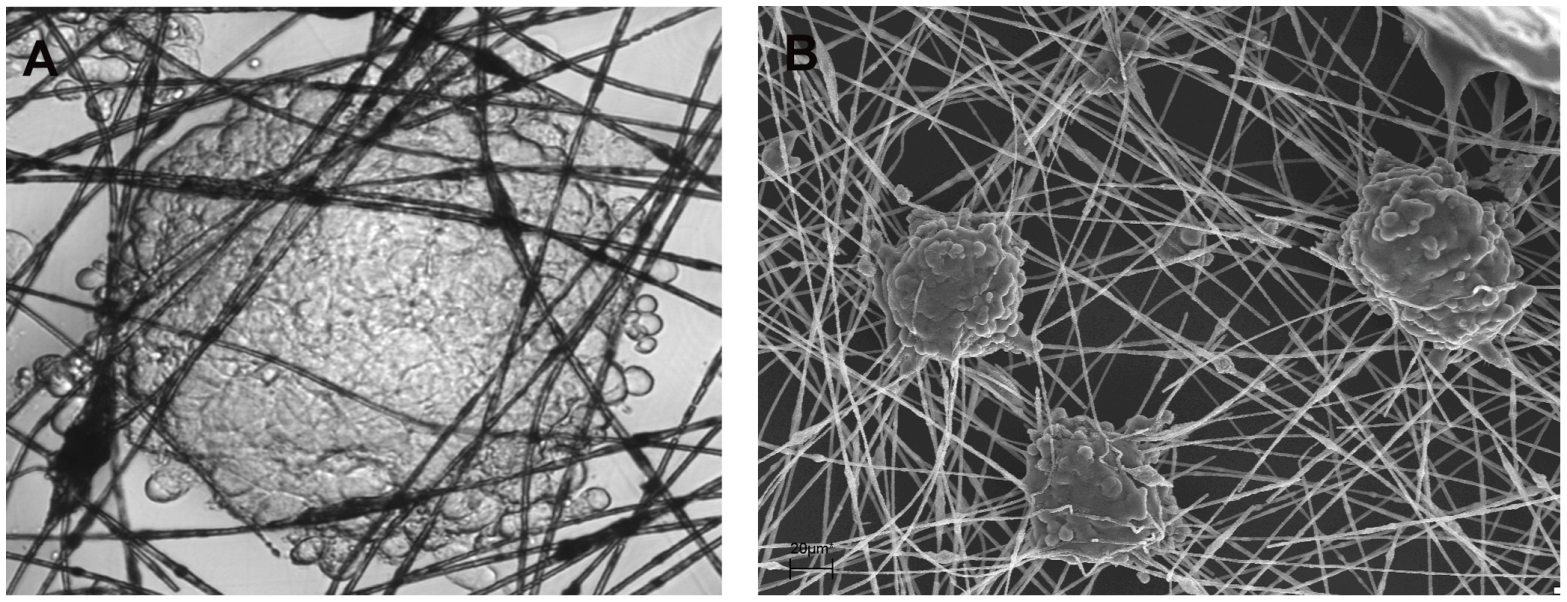
Cells growing on the scaffold visualized using light microscopy after 48 hours of incubation. Original magnification x200 (A). Scanning electron microscopy image of HeLa cells on the nanostructured grid fibers after 48 hours of incubation. Original magnification x100 (B).

The results of the light microscopy and scanning electron microscopy observation demonstrated that after 48 hours of culture on the nanostructured grids HeLa cells formed symmetrical and spherical aggregates. The size of cells ranged between 6 and 7 micrometers (Fig. 4).

**Figure 5:**
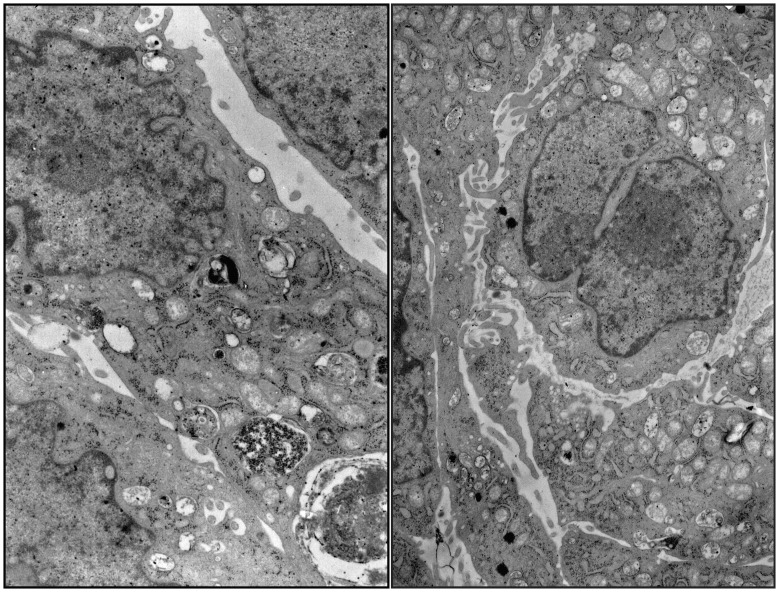
Transmission electron microscopy images of HeLa cells on the nanostructure grid fibers after 48 h culture. Original magnification x6000.

Detailed analysis of the cells grown for 48 hours on the nanostructured grids showed that there were no changes in the ultrastructure of HeLa cells. The cells were oval or elongated in shape with abundant microvilli and mostly euchromatic nuclei. The cytoplasm showed numerous intact organelles. Cisterns of the Golgi apparatus frequently contained electron-dense material in the form of laminae. The cells possessed numerous mitochondria with partially obliterated inner structure, sometimes presenting cristae. Some cells contained phagolysosomes with electron-dense content (Fig. 5).

## Discussion

Three-dimensional culture scaffolds assure a clearly better growth and proliferation of cells as they mimic the *in vivo* conditions. Moreover the cells cultured on 3D scaffolds have been found to develop a self-specific microenvironment [2],[13],[24]. Culture scaffolds of such properties can be used, for instance in regenerative medicine [2], [7], [9], [12], [22]–[23]. However, not all three-dimensional scaffolds are adequate for cell culture, as the building material of a scaffold must fulfill certain conditions [7], [10], [21], [25]. In particular it should allow for attachment of cells and their undisturbed growth and proliferation.

One of the most common among technologies used in production of nano- and microfibres from polymer solutions is electro-spinning. Studies confirm that this technique can be applied to obtain spatial scaffolds for cell cultures in *in vitro* conditions [20]–[21].

The electrospun three-dimensional nanostructured grids presented in this study were not only proven to be biocompatible but also suitable for cell culture. One of the advantages of these nanostructured grids is in their architecture in the form of nano- and microtubes. What is more in the process of electro-spinning these hollow fibers are being arranged randomly, forming an irregular network with micrometric distance between the tubes preserved. The electrospun capillaries possess nanopores which probably enhance the free flow of the culture medium. These features have favorable effects on cell attachment and their viability. It has also been shown that these properties may promote migration of the proliferating cells along the fibers or between the overlaying fiber layers [1].

XTT test results have demonstrated that the described scaffold is suitable for cell culture in 3D conditions. The intense and significant increase in cell mitochondrial activity was observed particularly 48 hours after seeding of cells on the grid. The observation has been additionally supported by results from fluorescent microscopy with the use of Hoechst 33342 and propidium iodide. It was demonstrated that within 24 hours of seeding, the cells attached to nanostructured grid fibers and formed spatial agglomerates, which in the course of culture formed multilayered structures.

It can be assumed that the process of cell seeding was accompanied by the selection of particular types of cells, which were capable of proliferation on the fibers and thereby accept the new supporting material. The problem which remains to be resolved involves our inability to calculate the precise number of cells deposited on the nanostructured grid in the first day of the culture. For this reason, the obtained values of mitochondrial activity have been related to results obtained for an empty nanostructured grid and then to each other.

The analysis of results obtained using a scanning electron microscope has shown that the cells deposited on nanostructured grids form protrusions, which may either anchor the cells at the site of deposition or allow their migration along the fibers. It may be expected that the anchored cells may form a barrier preventing further amoeba-like movements of the adjacent cells. This would explain the reason of the inward growth of cells and formation of the spherical spatial structures. The observed spherical shapes of cell agglomerates formed following 24 hours after cell deposition allow for local development of high density cultures on a limited area. Thus, the presented scaffold represents not only an excellent support for growing cells in 3D conditions but may also stabilize the environment of proliferating cells thanks to the specific microenvironment developed. Nevertheless, it should be noted that the above hypothesis requires verification using studies on interaction of cells with individual fibers.

The presence of pores in the described fibers not only promotes cell growth but also makes the grid transparent. The potential for cell observation and their growth monitoring using light microscope is an important advantage of the suggested method of 3D cultures. Such a feature eliminates the need for staining of cells with fluorescent markers or for their transfection using e.g. GFP. Moreover, the nanostructured grid-deposited cells may be used in studies on ultrastructure of cells in *in situ* conditions and thus the simple technique of preparing cultures for Spurr embedding may be here applied.

## Conclusions

A new solution for 3D cultures has been suggested, which is suitable for cell culture in conditions mimicking *in vivo* environment. The relatively high surface to volume ratio permits to load such a nanostructured grid with high number of cells and to grow them in conditions of high density or even overcrowding. Moreover, the suggested novel 3D support in many ways surpasses other similar culture approaches: the scaffolds are transparent and thus allow observation of viable cells in time; standard fluorescence or colorimetric methods such can be used for evaluation of cells. Finally, the cell nanostructured grid can be embedded in *in situ* conditions for ultrastructural studies.
